# Relationship Between Standing Long Jump Performance and Health-Related Indicators and Lifestyle Factors in Chilean Adolescents: A Cross-Sectional Study

**DOI:** 10.3390/healthcare14121744

**Published:** 2026-06-17

**Authors:** Felipe Montalva-Valenzuela, Eduardo Guzmán-Muñoz, Antonio Castillo-Paredes, Camila Tapia Gatica, Natalia Escobar Ruiz, Yeny Concha-Cisternas, Pablo Valdés-Badilla, Álvaro Farfán-Díaz, Exal Garcia-Carrillo

**Affiliations:** 1 Escuela de Entrenador en Actividad Física y Deporte, Facultad de Ciencias Humanas, Universidad Bernardo O’Higgins, Santiago 8370040, Chile; felipe.montalva@ubo.cl (F.M.-V.); natalia.escobar@ubo.cl (N.E.R.); 2 Escuela de Kinesiología, Facultad de Salud, Universidad Santo Tomás, Talca 3460000, Chile; eguzmanm@santotomas.cl (E.G.-M.); yenyconchaci@santotomas.cl (Y.C.-C.); 3 Pedagogía en Educación Física, Facultad de Educación, Universidad Autónoma de Chile, Talca 3460000, Chile; 4 Grupo AFySE, Investigación en Actividad Física y Salud Escolar, Escuela de Pedagogía en Educación Física, Facultad de Educación, Universidad de Las Américas, Santiago 8370040, Chile; acastillop85@gmail.com; 5 Instituto Nacional del Fútbol, Deporte y Actividad Física (INAF), Peñalolén 7930013, Chile; ctapia@inaf.cl; 6 Vicerrectoría de Investigación e Innovación, Universidad Arturo Prat, Iquique 1100000, Chile; 7 Department of Physical Activity Sciences, Faculty of Education Sciences, Universidad Católica del Maule, Talca 3480112, Chile; pvaldes@ucm.cl; 8 Sports Coach Career, Faculty of Life Sciences, Universidad Viña del Mar, Viña del Mar 2520000, Chile; 9 Departamento de Nutrición y Kinesiología, Carrera de Nutrición, Facultad de Ciencias de la Salud, Universidad de Tarapacá, Arica 1000000, Chile; alvarofarfandiaz@gmail.com; 10 Department of Physical Activity Sciences, Universidad de Los Lagos, Osorno 5290000, Chile

**Keywords:** standing long jump, adolescents, lifestyles, muscular fitness, health

## Abstract

**Background:** The standing long jump (SLJ) is a field-based test of lower-limb muscular fitness in youth and a proposed indicator of health-related physical fitness. However, evidence on its relationship with psychological, sleep, dietary, and lifestyle factors in adolescents remains limited, particularly in Latin America. Objective: To examine the association between SLJ performance and health-related indicators and lifestyle factors in Chilean adolescents. **Methods:** A cross-sectional study was conducted with 145 adolescents (16.02 ± 1.09 years; 99 males, 46 females) from a subsidized secondary school in Chile. SLJ performance, handgrip strength, body mass index (BMI), physical activity (PAQ-A), sleep quality (PSQI), dietary indices (HCI and UHCI), and psychological symptoms (DASS-21) were assessed. Associations were examined using Pearson’s correlation and multiple linear regression, with false discovery rate (FDR) correction for multiple comparisons. **Results:** SLJ performance was positively associated with handgrip strength (r = 0.59, *p* < 0.001) and physical activity (r = 0.42, *p* < 0.001), and negatively associated with BMI (r = −0.30, *p* = 0.001) and anxiety (r = −0.24, *p* = 0.011). Sleep quality showed a weak inverse association that was not significant after FDR correction. Dietary indices were not associated with SLJ performance. In the adjusted model, sex (β = 0.224, *p* = 0.013), BMI (β = −0.275, *p* < 0.001), handgrip strength (β = 0.435, *p* < 0.001), and physical activity (β = 0.229, *p* = 0.002) were independently associated with SLJ performance, explaining 57% of the variance (R^2^ = 0.57, adjusted R^2^ = 0.523). **Conclusions:** SLJ performance is mainly associated with muscular strength, physical activity, and BMI in Chilean adolescents, while psychological, dietary, and sleep variables are not independently associated after adjustment. These findings support SLJ as a practical indicator of muscular fitness, but not of overall health. Due to the cross-sectional design, causal inference is not possible.

## 1. Introduction

The standing long jump (SLJ) is a fundamental motor skill [1] strongly associated with success in various sports disciplines [2]. Furthermore, it is a complex action requiring a high level of coordination [3]. Beyond its relevance for sports performance, this test is used as an indicator of physical fitness in different age groups [4,5], including physical education classes [6], as it reflects muscular fitness [7] and, consequently, is indirectly associated with health-related outcomes [8]. In this context, field-based tests such as the SLJ have gained attention as practical tools for the early identification of health-related risk factors in youth [9].

The SLJ offers multiple advantages: it is a validated and reliable test in children and adolescents [10], as well as being practical, inexpensive, and easy to interpret. These characteristics make it particularly suitable for large-scale applications in school and community settings, where access to more complex clinical assessments may be limited. It can also be used as a marker of physical performance and as a predictor of athletic potential [11]. Furthermore, the National Academy of Medicine recommends this test as a measure of muscular fitness [12].

Although SLJ has been widely studied as a marker of muscular fitness, evidence regarding its relationship with psychological factors, sleep quality, and dietary habits in adolescents remains limited, particularly in Latin American populations. Moreover, most previous studies have focused primarily on anthropometric and physical fitness outcomes, while comparatively less attention has been given to broader lifestyle-related factors that may influence or be associated with SLJ performance during adolescence.

Muscular fitness is an important marker of health in young people [13] and comprises muscle strength, explosive power, and endurance [14]. Evidence shows positive associations with bone health and inverse associations with various risk factors, such as adiposity, cardiovascular disease, and metabolic disorders [15,16]. Furthermore, muscular fitness can improve insulin sensitivity in both healthy individuals and those at higher metabolic risk, reinforcing its relevance during growth and development [17].

Healthy lifestyle habits are key determinants of health, where positive changes can have a significant impact [18]. Therefore, it is essential to promote these habits from an early age, especially during adolescence [19]. Among the main habits associated with health are physical activity, sleep quality, healthy diet, and mental health, among others [20]. From a preventive healthcare perspective, the identification of simple indicators associated with these lifestyle factors may help support and guide early interventions aimed at reducing future cardiometabolic and mental health risks.

Schools represent an important setting for promoting physical fitness and healthy behaviors during adolescence [21,22]. Physical education classes and extracurricular physical activity opportunities may contribute to improvements in muscular fitness and overall health [23,24]. Consequently, identifying simple and accessible field-based indicators associated with these factors may facilitate health monitoring and support health promotion strategies in school settings.

Based on the above, it is relevant to understand how lifestyle habits are associated with performance in SLJ. Clarifying these relationships may contribute to the identification of accessible, field-based indicators that can be used in school and primary care contexts as part of early screening and health promotion strategies. These results could provide relevant information for health promotion during adolescence. In addition, simultaneously examining physical, behavioral, psychological, sleep-related, and dietary variables may help determine whether SLJ performance reflects a broad multidimensional health construct or is more specifically associated with physical fitness-related domains during adolescence. We hypothesized that better SLJ performance would be positively associated with muscular strength, physical activity levels, and healthier lifestyle patterns, and negatively associated with BMI, poorer sleep quality, and adverse psychological symptoms in adolescents. Therefore, this study aimed to analyze the relationship between the SLJ and health-related indicators and lifestyle factors in secondary school students in Chile.

## 2. Materials and Methods

### 2.1. Study Design

This quantitative, descriptive–correlational study employed a cross-sectional observational design [25]. The protocol was approved by the Ethics Committee of Universidad Santo Tomás (Approval No. 23-27) and conducted in accordance with the principles of the Declaration of Helsinki. This study was conducted in accordance with the Strengthening the Reporting of Observational Studies in Epidemiology (STROBE) guidelines [26].

### 2.2. Participants

Participants were recruited from a subsidized private secondary school in the Metropolitan Region of Chile. The final sample comprised 145 adolescents (99 males and 46 females) aged 14–18 years (mean age 16.02 ± 1.09). Recruitment was based on a convenience sampling strategy due to accessibility considerations. Inclusion criteria were (a) enrollment in secondary education, (b) voluntary participation with informed assent and written parental consent, and (c) absence of musculoskeletal injury or medical contraindication to perform physical fitness tests. Students who reported acute pain, injury, or physical limitations during the previous three months were excluded from participation.

### 2.3. Procedures

Data collection was organized into three categories: anthropometric, physical fitness, and lifestyle–psychological assessments. All evaluations were conducted in the school gymnasium under standardized environmental conditions (temperature 20–23 °C, morning hours, and with participants wearing sports attire). All physical assessments were administered by the same trained evaluator to ensure consistency across measurements. To reduce potential fatigue effects, testing sessions were scheduled on separate days with a minimum recovery period of 48 h between sessions.

On the first day, participants completed the anthropometric measurements and received a familiarization session, during which researchers explained and demonstrated all testing procedures. The second day was dedicated to physical fitness assessments, including the SLJ and maximal isometric handgrip strength (MIHS) tests. On the third day, participants completed the lifestyle and psychological questionnaires—PAQ-A (Physical Activity Questionnaire for Adolescents), PSQI (Pittsburgh Sleep Quality Index), HCI (Healthy Consumption Index), UHCI (Unhealthy Consumption Index), and DASS-21 (Depression, Anxiety, and Stress Scale–21)—individually in their classrooms under supervision, using their own mobile devices via Google Forms.

### 2.4. Standing Long Jump

Lower-limb explosive muscle strength was evaluated through the SLJ test, following standardized procedures previously described in the literature [27]. The SLJ has demonstrated adequate reliability and validity for assessing lower-body muscular power in children and adolescents, supporting its use as a field-based measure of muscular fitness [27]. All assessments were conducted indoors on a non-slip surface to ensure participant’s safety and measurement accuracy. Before testing, each participant completed a general warm-up lasting approximately 5 min, which included joint mobility and dynamic movements (e.g., bodyweight squats and light jogging) to reduce injury risk and optimize performance.

Participants began the test standing upright behind a clearly marked take-off line, with feet parallel and positioned shoulder-width apart. From this position, they were instructed to perform a maximal horizontal jump using both legs simultaneously, with free use of the arms to aid propulsion. The objective was to cover the greatest possible distance while maintaining balance upon landing. A trained evaluator measured the distance (in centimeters) from the take-off line to the point of heel contact closest to the start line using a flexible steel tape.

Each participant performed three attempts, interspersed with at least one minute of passive rest between jumps to minimize fatigue. The best performance among the three trials was recorded for analysis. During all SLJ, participants wore their own sports shoes, and evaluators provided verbal encouragement to ensure maximal effort.

### 2.5. Health-Related Indicators

Health-related indicators included BMI (body mass index), MIHS, and sleep quality. BMI was calculated as body weight (kg) divided by height squared (m^2^), using measurements obtained with a digital scale (Seca, Hamburg, Germany; accuracy 0.1 kg) and a wall-mounted stadiometer (Seca; accuracy 0.1 cm). Upper-limb muscle strength was assessed through MIHS, measured with a digital dynamometer (Camry EH101, Sensun Weighing Apparatus Group Ltd., Zhongshan, China). During the measurement, participants stood upright with the elbow flexed at 90°, the forearm in a neutral position, and the arm held close to the trunk without contact. The dynamometer handle was adjusted to fit each participant’s hand size, ensuring the second phalanx rested comfortably on the handle. Participants were then instructed to squeeze the dynamometer with maximal effort for approximately three seconds, while maintaining proper posture and avoiding compensatory movements. The test was performed using the dominant hand. Each participant completed three attempts with at least one minute of rest between trials, and the highest value (kg) was retained for analysis [28].

Sleep quality was evaluated using the PSQI [29], which assesses seven components related to sleep duration, latency, and disturbances. The PSQI consists of 19 self-administered items grouped into seven components: (i) subjective sleep quality, (ii) sleep latency, (iii) sleep duration, (iv) habitual sleep efficiency, (v) sleep disturbances, (vi) use of sleep medication, and (vii) daytime dysfunction. Each component is scored on a scale from 0 to 3, where higher scores indicate greater impairment. The global PSQI score, obtained by summing the seven components, ranges from 0 to 21 points. A total score ≤ 5 indicates good sleep quality, whereas scores > 5 reflect poor sleep quality or clinically relevant disturbances in sleep patterns. Although the PSQI was originally developed for adult populations, previous studies have demonstrated adequate reliability and validity in adolescent samples [29].

### 2.6. Lifestyle Factors

Lifestyle factors included physical activity, dietary patterns, and psychological well-being, which together reflect key components of adolescent health behavior.

Physical activity levels were assessed using the PAQ-A [30]. This self-administered instrument evaluates moderate-to-vigorous physical activity during the previous seven days across school, leisure, and sports contexts. Scores range from 1 (low activity) to 5 (high activity), with higher values indicating greater engagement in habitual physical activity.

Dietary patterns were analyzed using a semi-quantitative food frequency questionnaire that included common food items consumed by Chilean adolescents [31]. From these data, two indices were constructed to reflect the quality of eating habits: the HCI and the UHCI.

The HCI was calculated by summing the weekly frequency of consumption of food groups considered healthy, such as fruits, vegetables, legumes, fish, dairy products, and whole grains. The UHCI, on the other hand, represented the sum of the weekly frequency of intake of food groups classified as unhealthy, including sugar-sweetened beverages, fried foods, sweets, fast food, and processed snacks. Each item was scored on a scale from 0 (never or rarely consumed) to 4 (consumed daily), and the total scores were standardized so that higher values in the HCI denoted better diet quality, whereas higher values in the UHCI indicated poorer dietary quality. The selection and classification of food groups were based on a dietary quality framework previously proposed and validated in the Chilean population, adapted to national dietary guidelines [31]. This approach has demonstrated feasibility and construct validity as a population-level indicator of dietary behavior, rather than a clinical measure of nutritional intake.

Psychological well-being was assessed using the DASS-21 [32], which consists of 21 items distributed across three subscales with seven items each. Responses were rated from 0 (“Did not apply to me at all”) to 3 (“Applied to me very much or most of the time”), and average subscale scores were computed for each domain. Higher values reflect greater levels of stress, anxiety, or depressive symptoms.

### 2.7. Statistical Analysis

All statistical analyses were performed using GraphPad Prism version 9.0 (GraphPad Software, San Diego, CA, USA). Continuous variables were expressed as mean ± standard deviation (SD), and categorical variables, when applicable, as frequencies and percentages. Before performing inferential tests, the Shapiro–Wilk test was applied to verify the assumption of normality, and the Levene test was used to evaluate the homogeneity of variances between groups. Subsequently, independent samples *t*-tests were used to compare differences between males and females in anthropometric, physical fitness, behavioral, dietary, and psychological variables; when the assumption of homogeneity of variance was violated, Welch’s correction was applied. In addition, the correlations between SLJ performance and health-related indicators and lifestyle factors (BMI, MIHS, physical activity level, sleep quality, HCI, UHCI, stress, anxiety, and depression) was examined using Pearson’s correlation coefficient (r), both in the total sample and stratified by sex. Correlation strength was interpreted as small (|r| = 0.10–0.29), moderate (|r| = 0.30–0.49), or large (|r| ≥ 0.50). To reduce the risk of type I error due to multiple testing, *p*-values obtained from the correlation analyses were additionally adjusted using the Benjamini–Hochberg false discovery rate (FDR) procedure, with statistical significance established at q < 0.05.

A post hoc statistical power analysis was conducted to evaluate whether the available sample size was sufficient to detect correlations between SLJ performance and health-related indicators and lifestyle factors. Considering the total sample size of 145 participants, a two-tailed α level of 0.05, and Pearson’s correlation analysis, the study had approximately 89% power to detect a moderate correlation of r = 0.30 and >99% power to detect a large correlation of r = 0.50. However, the statistical power to detect small correlations, such as r = 0.20, was limited. Therefore, the study was considered adequately powered to detect moderate-to-large associations, whereas small associations should be interpreted with caution.

To further examine the independent associations between SLJ performance and health-related indicators and lifestyle factors, a fully adjusted multiple linear regression model was performed using SLJ performance as the dependent variable. Sex, age, BMI, MIHS, physical activity level, sleep quality, HCI, UHCI, stress, anxiety, and depression were included as independent variables. Regression results were reported as unstandardized coefficients (B), standard errors (SE), standardized regression coefficients (β), 95% confidence intervals (95% CI), and *p*-values. Multicollinearity among the independent variables included in the multiple linear regression model was assessed using tolerance values and variance inflation factors (VIF). Tolerance values <0.10 and/or VIF values >10.0 were considered indicative of severe multicollinearity. Since all predictors showed acceptable tolerance and VIF values, no variable was excluded from the final model due to multicollinearity.

All tests were two-tailed, and a *p*-value < 0.05 was considered statistically significant.

## 3. Results

Table 1 presents the descriptive characteristics of the participants. The total sample comprised 145 adolescents (99 males and 46 females) with a mean age of 16.02 ± 1.09 years. Males showed higher mean values for weight (64.81 ± 8.73 kg) and height (171.22 ± 6.03 cm) compared to females (62.37 ± 9.06 kg and 165.81 ± 8.57 cm, respectively). Conversely, females presented a slightly higher BMI (22.31 ± 2.85 kg/m^2^) than males (22.09 ± 2.71 kg/m^2^).

Table 2 presents the descriptive statistics for physical fitness, health-related indicators, and lifestyle factors. The comparison between males and females revealed that males exhibited greater performance in the SLJ (194.3 ± 25.5 vs. 155.8 ± 23.3 cm; *p* < 0.001) and MIHS (36.0 ± 7.3 vs. 26.2 ± 4.5 kg; *p* < 0.001) compared to females. Similarly, males reported higher levels of physical activity (2.71 ± 0.74 vs. 2.28 ± 0.72; *p* = 0.005) and better sleep quality (lower PSQI scores: 6.16 ± 3.45 vs. 8.20 ± 4.11; *p* = 0.012).

Regarding dietary patterns, males had a slightly higher HCI (2.74 ± 0.99 vs. 2.34 ± 0.92; *p* = 0.045), while no significant differences were found in the UHCI (*p* = 0.380). In the psychological domain, females reported higher levels of stress (11.22 ± 4.07 vs. 8.91 ± 4.08; *p* = 0.006) and anxiety (9.58 ± 5.22 vs. 6.31± 4.13; *p* = 0.002), whereas no significant difference was observed in depression (*p* = 0.159).

Table 3 shows the correlations between SLJ performance and the health-related indicators and lifestyle factors. In the total sample, SLJ performance was positively associated with MIHS (r = 0.59, *p* < 0.001) and physical activity levels (r = 0.42, *p* < 0.001), while it was negatively correlated with BMI (r = −0.30, *p* = 0.001) and anxiety (r = −0.24, *p* = 0.011). Sleep quality showed a weak negative correlation with SLJ performance in the unadjusted analysis (r = −0.16, *p* = 0.042); however, this association was no longer significant after false discovery rate correction and was not maintained in the adjusted regression model.

When analyzed by sex, MIHS remained positively associated with SLJ performance in both males (r = 0.37, *p* = 0.001) and females (r = 0.45, *p* = 0.006). Among females, physical activity was also strongly associated with SLJ performance (r = 0.55, *p* < 0.001). Although BMI and physical activity showed nominally significant correlations in males before correction for multiple testing, these associations were no longer significant after false discovery rate adjustment. No significant correlations were found between SLJ and dietary indices (HCI, UHCI) or psychological variables such as stress, anxiety, or depression when analyzed separately by sex.

**Figure 1:**
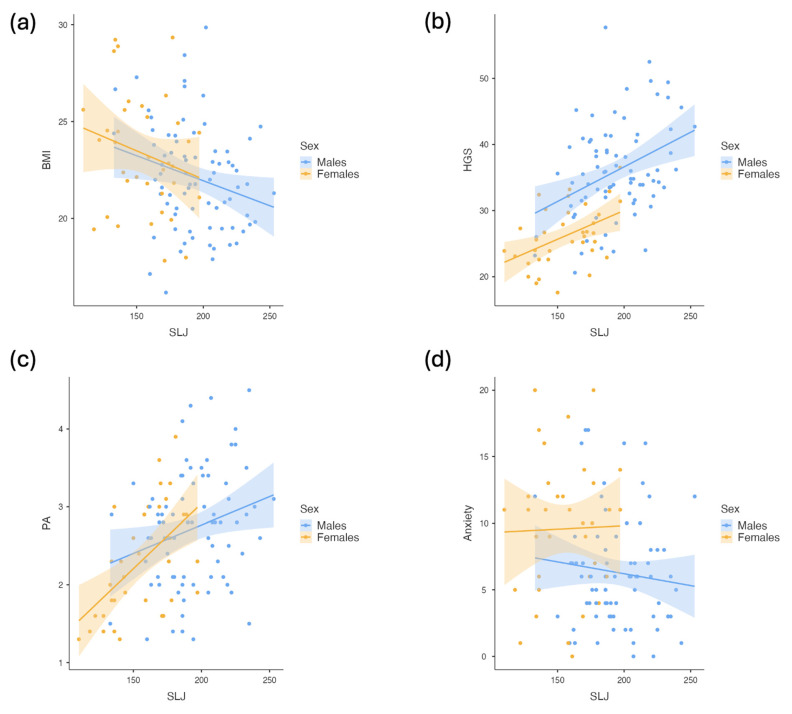
Sex-stratified relationships between SLJ performance and selected health-related variables showed significant correlations. Panels show relationships between SLJ and (**a**) body mass index (BMI), (**b**) maximal isometric handgrip strength (MIHS), (**c**) physical activity (PA), and (**d**) anxiety scores. Solid lines represent linear regression fits with 95% confidence intervals.

Figure 1 illustrates the relationship between SLJ performance and the variables that showed significant correlations in the total sample. Visual inspection suggests an inverse relationship between SLJ and BMI, as well as a direct relationship with MIHS and physical activity levels. A negative trend between SLJ and anxiety scores was also observed in the total sample. Sex-specific regression lines illustrate differences in the dispersion and magnitude of these relationships, particularly for physical activity and BMI.

The overall regression model was statistically significant and explained 57.0% of the variance in SLJ performance (R^2^ = 0.570; adjusted R^2^ = 0.523; *p* < 0.001). As shown in Table 4, sex, BMI, MIHS, and physical activity level were independently associated with SLJ performance. Specifically, male sex was positively associated with higher SLJ performance (B = 14.644; 95% CI: 3.118 to 26.171; *p* = 0.013). BMI showed a significant negative association with SLJ performance (B = −2.932; 95% CI: −4.474 to −1.391; *p* < 0.001), indicating that higher BMI was associated with lower jump performance. In contrast, MIHS was positively associated with SLJ performance (B = 1.668; 95% CI: 1.002 to 2.333; *p* < 0.001), as was physical activity level assessed through the PAQ-A (B = 9.216; 95% CI: 3.393 to 15.039; *p* = 0.002).

No significant independent associations were observed between SLJ performance and age, sleep quality, HCI, UHCI, stress, anxiety, or depression after adjustment for the remaining variables. Variance inflation factor values ranged from 1.13 to 2.55, indicating no evidence of problematic multicollinearity among the predictors.

## 4. Discussion

This study aimed to analyze the relationship between the SLJ and health-related indicators and lifestyle factors in secondary school students in Chile. An important finding of the present study is that, although SLJ performance was associated with several health-related indicators and lifestyle factors in the bivariate analyses, only sex, BMI, muscular strength, and physical activity remained independently associated with SLJ performance after adjustment for potential confounders. This suggests that SLJ performance may primarily reflect physical fitness-related characteristics rather than broader psychological, nutritional, or sleep-related domains. Specifically, SLJ performance was positively associated with muscular strength and physical activity level, and negatively associated with BMI, anxiety, and sleep quality in the bivariate analyses. However, the associations with anxiety and sleep quality were no longer significant after adjustment, indicating that these relationships may be explained by other physical fitness-related factors. These findings should be interpreted as associations rather than causal relationships due to the cross-sectional design.

The positive relationship between the SLJ and the MIHS is consistent with the findings of Nakai et al. [33], who suggest that performance on this test does not depend solely on lower limb muscle strength, but that upper limb muscle strength must also be considered. Although the underlying mechanisms were not assessed in the present study, this relationship could be explained by coordinated force production between the upper and lower limbs or, more generally, by a higher level of overall physical fitness in the students assessed. However, these interpretations remain speculative and should be confirmed in studies with longitudinal or experimental designs.

On the other hand, the negative relationship between SLJ and BMI is consistent with previous evidence suggesting that body mass relative to height may influence physical performance in adolescents, supporting previous evidence indicating that greater body mass relative to height may negatively influence jumping performance in adolescents [34]. Likewise, the inverse association observed between SLJ and anxiety in the bivariate analyses may indicate a potential relationship between muscular fitness and psychological well-being, which is consistent with previous evidence reporting inverse associations between physical fitness and anxiety symptoms in adolescents [35]. However, this association was no longer significant in the fully adjusted model, suggesting that the relationship observed in the present study may be influenced by other physical fitness-related factors or potential confounders.

Collectively, the observed bivariate relationships between SLJ, anxiety, and sleep quality suggest that psychological and sleep-related factors may be linked to muscular fitness during adolescence. However, the absence of independent associations after adjustment indicates that these factors are likely less strongly related to SLJ performance than physical fitness-related variables such as muscular strength, BMI, and physical activity. Nevertheless, these findings do not allow conclusions regarding directionality or causality. These results are consistent with previous studies that have shown a relationship between muscle performance and better sleep quality in adolescents [36]. Although sleep quality showed a weak bivariate association with SLJ performance in the unadjusted analysis, this relationship was no longer significant after correction for multiple testing and was not maintained after adjustment for potential confounders, suggesting that the observed association should be interpreted cautiously. Overall, the findings support the relevance of muscular fitness and physical activity as important factors associated with SLJ performance during adolescence.

Additionally, sex differences were observed in some lifestyles, with males reporting higher physical activity levels and better sleep quality compared to females. While these findings are consistent with previous evidence describing higher levels of physical activity in males [37], they should be interpreted with caution due to the lower female representation in the sample. In this context, the higher physical activity level reported by males, along with better performance on the SLJ, could help explain the better sleep quality observed in this group.

Regarding dietary habits, males showed a slightly higher HCI, with no difference in the UHCI. This result contrasts with previous studies that indicate a greater tendency for females to choose healthier foods [38]. However, these discrepancies could be explained by cultural, contextual, and economic differences, considering that the available studies have been conducted in populations with different socioeconomic realities, which suggests the need to further explore this relationship. Importantly, no significant associations were observed between dietary indices and SLJ performance. Therefore, the role of dietary habits in explaining muscular fitness within the present sample remains unclear and warrants further investigation using more comprehensive dietary assessments.

In the psychological sphere, females reported higher levels of stress, a finding consistent with previous research [39,40] and highlighting the importance of considering sex differences in the analysis of mental health during adolescence. Future studies should consider contextual factors such as family support, socioeconomic status, biological maturation, and access to recreational spaces, as these variables may influence both lifestyle behaviors and physical fitness during adolescence.

### 4.1. Limitations

Among the study’s limitations is the selection of participants, which was conducted using convenience sampling due to the ease of access to the sample and recruitment from a single school. In addition, the relatively small sample size constitutes a key limitation, as it may reduce statistical power and limit the generalizability of the findings to broader adolescent populations. Furthermore, although the sample size met conventional minimum recommendations for multiple linear regression analyses, the number of predictors included in the fully adjusted regression model relative to the available sample size may have increased the risk of model overfitting and reduced the stability of some regression estimates. Therefore, the results of the multivariable analyses should be interpreted with caution and confirmed in larger independent samples. There was also an imbalance between male and female participants, with lower female representation further limiting the ability to draw robust sex-specific conclusions and potentially introducing bias in subgroup analyses. Together, these factors substantially reduce the generalizability of the findings. Furthermore, relevant variables such as socioeconomic status, pubertal maturation, and biological maturation level were not assessed, despite their potential influence on physical performance and health-related outcomes during adolescence. Additionally, BMI was the only anthropometric indicator used in the present study. Although BMI is widely applied in epidemiological research, it does not distinguish between fat mass, muscle mass, or growth-related changes during adolescence.

The use of self-report questionnaires can also introduce bias, as reading fatigue, attention, concentration, reading comprehension, or other external factors can influence the results. Furthermore, it is important to mention that external physical fatigue among the participants could not be controlled. Although they were informed of and asked to take protective measures within their weekly routine and sporting activities, there is no evidence to support their compliance. Additionally, footwear was not standardized during SLJ assessments, as participants used their own sports shoes, which may have influenced jumping mechanics, stability, or traction.

Moreover, the cross-sectional design represents an important limitation, as it precludes establishing causal relationships or determining the directionality of the observed associations. Finally, and most importantly, relationships do not establish causation; therefore, we recommend interpreting these results with caution. Taken together, these limitations suggest that the findings should be interpreted as preliminary and hypothesis-generating rather than definitive.

### 4.2. Future Lines of Investigation

Ideally, the next step would be to find a method that allows for more random selection and even a larger sample size. It is also important to encourage female participation and create a sample with a more balanced representation of men and women. It would also be interesting to consider some socioeconomic factors, place of residence, available spaces and open areas for recreation, etc. Including questionnaires on physical self-perception and quality of life would be beneficial. Finally, it would be interesting to broaden the scope and consider body composition rather than weight, since weight can be a limiting factor that does not account for muscle mass or body fat percentage. In the same line, future research should also include indicators of biological maturation (e.g., Tanner staging or maturity offset), given their known influence on physical performance during adolescence.

### 4.3. Practical Recommendations

The findings of this study suggest that the SLJ may have potential as a simple and low-cost field-based indicator of muscular fitness in adolescents. However, its use as a standalone tool for assessing broader health domains (e.g., nutritional status or mental health) should be approached with caution, given the cross-sectional design and sample size.

Given its ease of application in school settings, the SLJ could be considered as part of a broader assessment battery within physical education or health promotion programs, rather than as a diagnostic or screening tool on its own.

Furthermore, the observed associations between SLJ performance and variables such as physical activity, anxiety, and sleep quality highlight the potential relevance of muscular fitness within a multidimensional health framework. Nevertheless, these findings should not be interpreted as evidence of causal effects, and further research is needed before translating them into specific intervention strategies.

## 5. Conclusions

The present findings suggest that SLJ performance is primarily associated with physical fitness-related factors, particularly muscular strength, BMI, and physical activity levels in Chilean adolescents. Although some psychological and sleep-related variables showed weak bivariate associations, these relationships were not maintained after adjustment for potential confounders. Therefore, SLJ may serve primarily as a practical field-based indicator of muscular fitness rather than a broader multidimensional health marker.

More specifically, SLJ performance showed a positive relationship with handgrip strength and a negative relationship with body mass index, anxiety, and, to a lesser extent, sleep quality. In the fully adjusted model, sex, body mass index, handgrip strength, and habitual physical activity remained independently associated with SLJ performance. Differences were also observed between sexes, with males reporting higher levels of physical activity, better sleep quality, and a higher healthy eating index, while females reported higher stress levels.

However, given the cross-sectional design and relatively small sample size, these findings should be interpreted with caution, as no causal inferences can be drawn and generalizability may be limited.

## Figures and Tables

**Table 1:** Descriptive characteristics of the participants (Mean ± SD).

Variable	Total (n = 145)	Males (n = 99)	Females (n = 46)
Age (years)	16.02 ± 1.09	16.07 ± 1.10	15.89 ± 1.02
Weight (kg)	62.84 ± 9.02	64.81 ± 8.73	62.37 ± 9.06
Height (cm)	167.19 ± 8.42	171.22 ± 6.03	165.81 ± 8.57
BMI (kg/m^2^)	22.48 ± 2.86	22.09 ± 2.71	22.31 ± 2.85

BMI: body mass index.

**Table 2:** Descriptive statistics of physical fitness, health-related indicators, and lifestyle factors (Mean ± SD).

Variable	Total (n = 145)	Males (n = 99)	Females (n = 46)
SLJ (cm)	182.09 ± 29.10	194.26 ± 25.45	155.75 ± 23.30
MIHS (kg)	32.34 ± 7.71	36.02 ± 7.27	26.16 ± 4.48
PAQ-A	2.56 ± 0.76	2.71 ± 0.74	2.28 ± 0.72
Sleep quality (PSQI score)	6.78 ± 3.82	6.16 ± 3.45	8.20 ± 4.11
HCI	2.60 ± 0.97	2.74 ± 0.99	2.34 ± 0.92
UHCI	2.28 ± 0.90	2.20 ± 0.88	2.42 ± 0.93
Stress (score)	9.76 ± 3.85	8.91 ± 4.08	11.22 ± 4.07
Anxiety (score)	7.61 ± 4.70	6.31 ± 4.13	9.58 ± 5.22
Depression (score)	7.93 ± 4.98	7.35 ± 4.70	8.86 ± 5.47

SLJ = standing long jump; MIHS = maximal isometric handgrip strength; PAQ-A = Physical Activity Questionnaire Score; HCI = Healthy Consumption Index; UHCI = Unhealthy Consumption Index.

**Table 3:** Correlations between SLJ performance and health-related indicators and lifestyle factors in adolescents.

Variable	Statistic	Total (n = 145)	Males (n = 99)	Females (n = 46)
BMI	R	−0.304 **	−0.244 *	−0.224
	P	0.001	0.033	0.189
	q (FDR)	0.009	0.059	0.340
MIHS	R	0.591 ***	0.366 **	0.453 **
	P	<0.001	0.001	0.006
	q (FDR)	<0.001	0.009	0.027
PAQ-A	R	0.415 ***	0.249 *	0.546 ***
	P	<0.001	0.029	<0.001
	q (FDR)	<0.001	0.059	<0.001
Sleep quality (PSQI score)	R	−0.164	−0.006	−0.088
	P	0.042 *	0.960	0.615
	q (FDR)	0.068	0.994	0.814
HCI	R	0.072	−0.173	0.274
	P	0.449	0.132	0.106
	q (FDR)	0.629	0.257	0.238
UHCI	R	−0.036	0.061	−0.073
	P	0.708	0.599	0.671
	q (FDR)	0.814	0.808	0.814
Stress	R	−0.153	0.001	−0.005
	P	0.106	0.994	0.975
	q (FDR)	0.238	0.994	0.994
Anxiety	R	−0.239 *	−0.110	0.023
	P	0.011	0.342	0.894
	q (FDR)	0.050	0.577	0.994
Depression	R	−0.106	0.014	−0.116
	P	0.262	0.903	0.500
	q (FDR)	0.442	0.994	0.675

Values represent Pearson’s correlation coefficients (r) and corresponding *p*-values. BMI = body mass index; MIHS = maximal isometric handgrip strength; PAQ-A = Physical Activity Questionnaire Score; HCI = Healthy Consumption Index; UHCI = Unhealthy Consumption Index. * *p* < 0.05; ** *p* < 0.01; *** *p* < 0.001. False discovery rate (Benjamini–Hochberg) correction was applied to the correlation analyses. Adjusted *p*-values are reported as q-values.

**Table 4:** Independent associations of health-related indicators and lifestyle factors with SLJ performance in adolescents: a fully adjusted multiple linear regression model.

Variable	B	SE	β	95% CI Lower	95% CI Upper	*p*-Value
Sex, male = 1	14.644	5.811	0.224	3.118	26.171	0.013
Age (years)	−0.282	2.044	−0.010	−4.336	3.772	0.891
BMI (kg/m^2^)	−2.932	0.777	−0.275	−4.474	−1.391	<0.001
MIHS (kg)	1.668	0.335	0.435	1.002	2.333	<0.001
PAQ-A	9.216	2.935	0.229	3.393	15.039	0.002
Sleep quality (PSQI score)	−0.185	0.639	−0.023	−1.452	1.082	0.773
HCI	−1.408	2.156	−0.045	−5.684	2.869	0.515
UHCI	−0.913	1.887	−0.036	−4.657	2.830	0.629
Stress (score)	0.283	0.723	0.039	−1.151	1.718	0.696
Anxiety (score)	−0.321	0.673	−0.050	−1.656	1.013	0.634
Depression (score)	0.320	0.567	0.052	−0.805	1.446	0.573

B = unstandardized regression coefficient; SE = standard error; β = standardized regression coefficient; CI = confidence interval; BMI = body mass index; MIHS = maximal isometric handgrip strength; PAQ-A = Physical Activity Questionnaire for Adolescents; PSQI = Pittsburgh Sleep Quality Index; HCI = Healthy Consumption Index; UHCI = Unhealthy Consumption Index. Sex was coded as 0 = female and 1 = male.

## Data Availability

The data presented in this study are available from the corresponding author upon reasonable request. The data are not publicly available due to ethical restrictions and privacy concerns involving minors.

## Abbreviations

The following abbreviations are used in this manuscript:

SLJ	Standing Long Jump
BMI	Body Mass Index
MIHS	Maximal Isometric Handgrip Strength
PAQ-A	Physical Activity Questionnaire for Adolescents
PSQI	Pittsburgh Sleep Quality Index
HCI	Healthy Consumption Index
UHCI	Unhealthy Consumption Index
DASS-21	Depression, Anxiety, and Stress Scale–21
